# Exploring the Nexus of Nicotine Dependence and Schizophrenia in a Clinical Case Study

**DOI:** 10.1192/j.eurpsy.2025.931

**Published:** 2025-08-26

**Authors:** S. Ben Aissa, N. Chaima, R. Khouloud, L. Amine, M. Wahid

**Affiliations:** 1 Psychiatry D, Razi Hospital, Mannouba, Tunisia

## Abstract

**Introduction:**

Studies in the general population and those based on clinical assessments of individuals with schizophrenia have shown a high degree of overlap between schizophrenia and substance-related disorders.

The prevalence of substance abuse throughout life is so common that the likelihood of a specific link inevitably arises. Various hypotheses have been proposed to explain the high comorbidity between schizophrenia and substance abuse, making these patients challenging to manage due to potential pharmacological interactions between the substances they consume and neuroleptic medications.

**Objectives:**

To explore the nexus between nicotine dependence and schizophrenia through a case report and a review of the literature.

**Methods:**

We discuss a 40-year-old divorced man, father of one daughter, unemployed, with a history of psychiatric follow-up in private care for seven months before admission for untreated schizophrenia, and polyaddiction to substances including tobacco, cannabis, and cocaine.

He was transferred to our facility for management of behavioral disorders and aggression with a refusal of oral treatment. The patient had been incarcerated in France for four years for assault with a bladed weapon. He was admitted to our emergency psychiatric service with his brother due to arson within his residence where he lives alone. Upon admission, the patient was well-oriented in time and space, with neglected hygiene, easy contact, neutral facial expressions, sad mood, blunted affect, motor instability, and a dissociative syndrome: irrelevant responses, tangential speech, and persecution delusions without a specific persecutor, confirming his behavioral disorder and trivializing it with impaired judgment and no suicidal ideations.

**Results:**

The patient was hospitalized and underwent a complete blood test and ECG, both of which returned normal results, and was treated with olanzapine 10 mg/day and chlorpromazine 200 mg/day. During his hospitalization, he made several attempts to start fires within the ward, explaining his actions as a constant desire to inhale smoke to relieve discomfort, and he experienced a craving for smoke. After a month in our facility, and following the resolution of the dissociative and delusional syndromes, we scheduled a leave for the patient to assess clinical improvement. Upon returning from leave, the patient expressed multiple persecutory delusions towards family members who prevented him from starting a fire in his garden, which he considered necessary for his well-being.

**Conclusions:**

Comorbidity between addiction and schizophrenia is very common in our social context, and management should be simultaneous, without neglecting the importance of family support.

**Disclosure of Interest:**

None Declared

